# Degradation of Orange G Using PMS Triggered by NH_2_-MIL-101(Fe): An Amino-Functionalized Metal–Organic Framework

**DOI:** 10.3390/molecules29071488

**Published:** 2024-03-27

**Authors:** Lijie Mo, Guangzhou Chen, Hua Wang

**Affiliations:** 1 Anhui Key Laboratory of Environmental Pollution Control and Waste Resource Utilization, Anhui Jianzhu University, Hefei 230601, China; 2 Anhui Key Laboratory of Water Pollution Control and Waste Water Recycling, Anhui Jianzhu University, Hefei 230601, China; 3 School of Environment and Energy Engineering, Anhui Jianzhu University, Hefei 230601, China; 4 Anhui Research Academy of Ecological Civilization, Anhui Jianzhu University, Hefei 230601, China; 5 Gansu Tobacco Industry Company Limited, Lanzhou 730050, China

**Keywords:** Orange G, peroxymonosulfate, metal–organic frameworks, NH_2_-MIL-101(Fe), advanced oxidation

## Abstract

As an azo dye, OG has toxic and harmful effects on ecosystems. Therefore, there is an urgent need to develop a green, environmentally friendly, and efficient catalyst to activate peroxymonosulfate (PMS) for the degradation of OG. In this study, the catalysts MIL-101(Fe) and NH_2_-MIL-101(Fe) were prepared using a solvothermal method to carry out degradation experiments. They were characterized by means of XRD, SEM, XPS, and FT-IR, and the results showed that the catalysts were successfully prepared. Then, a catalyst/PMS system was constructed, and the effects of different reaction systems, initial pH, temperature, catalyst dosing, PMS concentration, and the anion effect on the degradation of OG were investigated. Under specific conditions (100 mL OG solution with a concentration of 50 mg/L, pH = 7.3, temperature = 25 °C, 1 mL PMS solution with a concentration of 100 mmol/L, and a catalyst dosage of 0.02 g), the degradation of OG with MIL-101(Fe) was only 36.6% within 60 min; as a comparison, NH_2_-MIL-101(Fe) could reach up to 97.9%, with a reaction constant *k* value of 0.07245 min^−1^. The NH_2_-MIL-101 (Fe)/PMS reaction system was able to achieve efficient degradation of OG at different pH values (pH = 3~9). The degradation mechanism was analyzed using free-radical quenching tests. The free-radical quenching tests showed that SO_4_^•−^, •OH, and ^1^O_2_ were the main active species during the degradation of OG.

## 1. Introduction

With the rapid development of industry, dyes are often detected in wastewater, and most of these dyes have toxic, carcinogenic, and mutagenic effects [1]. Orange G, a typical azo dye, was used extensively in the paper, plastic, leather, and textile industries [2,3]. It is non-biodegradable, tenacious, and difficult to break down. Orange G in wastewater can produce harmful and toxic by-products through hydrolysis, oxidation, or other chemical processes, endangering both people and ecosystems [4]. The common removal methods for the dye currently include adsorption, coagulation–flocculation, membrane filtration, and advanced oxidation techniques [5,6,7,8].

Due to their high efficiency, green and low energy consumption, advanced oxidation techniques based on sulfate radicals (SR-AOPs) are widely utilized for the removal of organic contaminants that are difficult to decompose in bodies of water [9,10,11]. PMS is a common oxidant, and its asymmetric molecular structure facilitates its activation and produces transient radicals with a high oxidizing capacity and quick response rate [12,13]. PMS frequently needs to be activated in order to be effective, since it does not release enough oxidizing radicals on its own to break down organic contaminants [14]. Currently, the available techniques for the catalysis of PMS include electrical activation [15], photoactivation [16], and material activation [17], which all involve sulfate radical production. Van et al. [18] successfully synthesized a well-crystallized, high-purity magnetic MnFe_2_O_4_/α-MnO_2_ hybrid using the hydrothermal method, and the degradation of OG reached 96.8% by activating PMS within 30 min. Using the solvothermal method, Pu et al. [19] synthesized the catalyst MIL-53(Fe)-A; under optimal reaction conditions, the experiment results showed that the degradation rate of OG reached 93.7% within 180 min.

With their high specific surface area, porousness, and flexible structure, Fe-MOFs are a family of crystalline porous materials that are generated by iron ions or iron clusters linked with organic ligands by ligand bonding [20]. They are widely employed in adsorption [21], gas storage [22], and catalysis [23]. Because of its good catalytic characteristics, MIL-101(Fe) could increase the degradation effectiveness of PMS for pollutants through non-homogeneous phase catalysis and accelerate the transformation rate of Fe(III) to Fe(II) in MOFs through functional group alteration [24]. Additionally, it was anticipated that it could solve the issues of the low water stability of MOFs and a number of active sites being restricted [25].

Because the amino group (-NH_2_) can boost the number of active sites and hasten the rate of electron transfer, amino-functionalized MOFs have better catalytic and adsorption qualities than pure MOFs [26]. In order to degrade amaranth red, a toxic azo dye in water, Zhang et al. [27] prepared the metal–organic skeleton NH_2_-MIL-101(Fe) using the solvothermal method and showed that amino-functional MOFs could activate oxone steadily and efficiently. Liu et al. [28] added amino groups to MOFs, and the results indicated that adding amino groups could provide more effective adsorption sites in order to remove phosphate from water.

By adding amino functional groups, MOF catalyst NH_2_-MIL-101(Fe) with good catalytic performance was prepared in order to address the issue of the low activation performance of MIL-101(Fe). Target pollutant OG was chosen, and the effects of various reaction systems, temperature, initial pH, catalyst dosing, PMS dosing, and anionic interference on the degradation of OG were studied; UV full-band scanning and free-radical quenching tests were used to analyze the mechanism; and the material’s stability was examined.

## 2. Results and Discussion

### 2.1. Catalyst Characterization

**Figure 1:**
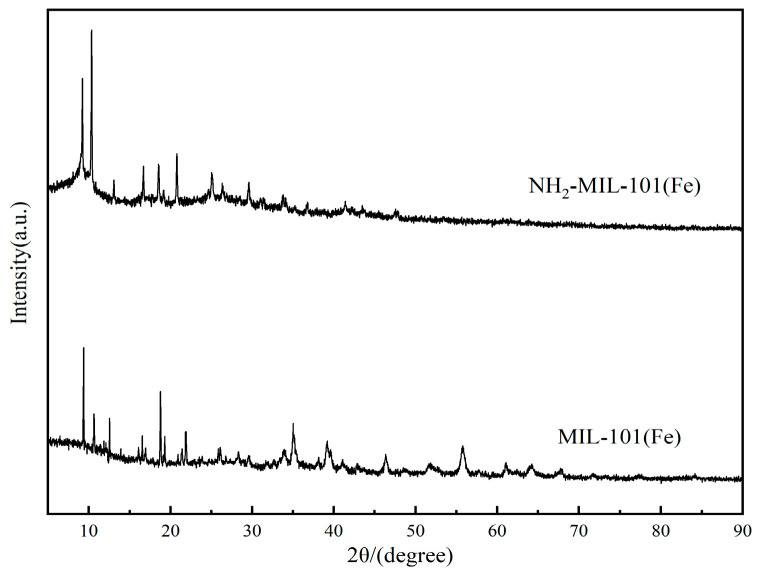
XRD patterns of NH_2_-MIL-101(Fe) and MIL-101(Fe).

As seen in Figure 1, the XRD patterns of the two iron-based MOFs displayed the distinctive peaks of the respective structures. The characteristic peaks appearing at 2θ = 9.2, 10.4, 13.1, 16.7, and 18.6 were essentially the same as the XRD patterns in the literature [29]. And, this indicated the successful preparation of NH_2_-MIL-101(Fe).

**Figure 2:**
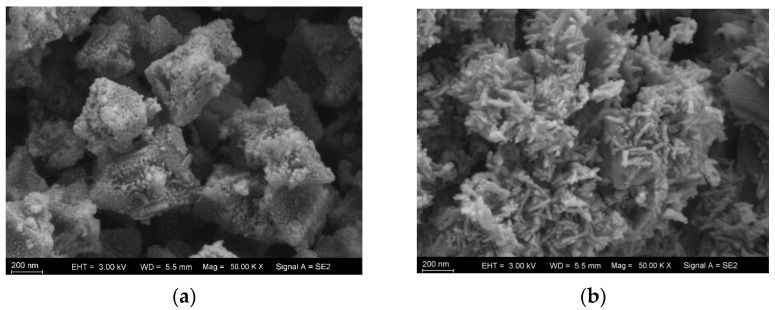
SEM images for (**a**) NH_2_-MIL-101(Fe) and (**b**) MIL-101(Fe).

Using scanning electron microscopy (SEM), the morphological characteristics of the materials MIL-101(Fe) and NH_2_-MIL-101(Fe) were observed. As illustrated in Figure 2, both materials displayed irregular polyhedral shapes, and the addition of amino functional groups changed the crystals’ shapes, demonstrating the successful addition of amino functional groups.

**Figure 3:**
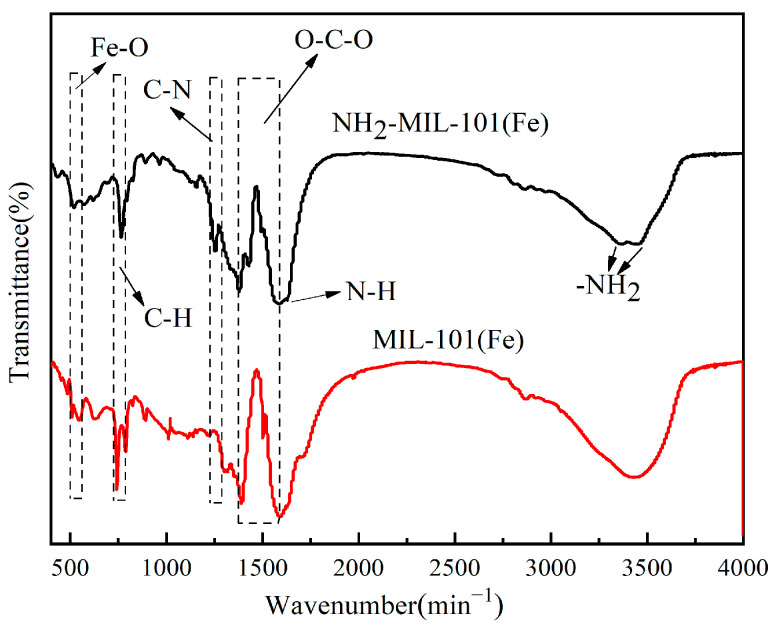
FT-IR images of NH_2_-MIL-101(Fe) and MIL-101(Fe).

The FT-IR spectra of the two MOF catalysts were shown in Figure 3. The peak at 550 cm^−1^ corresponded to the single bond vibration of Fe-O in MOFs [30]; the absorption peak at 761 cm^−1^ corresponded to the bending vibration of C-H; and the characteristic peaks around 1600–1300 cm^−1^ originate from the symmetrical and asymmetrical stretching vibrations of O-C-O [31]. The absorption peaks located at 1251 cm^−1^ and 1628 cm^−1^ corresponded to ν(C-N) and δ(N-H) [32], respectively, and the peaks at 3357 cm^−1^ and 3442 cm^−1^ were due to the stretching vibration of -NH_2_ [33], which confirmed the presence of amino functional groups.

**Figure 4:**
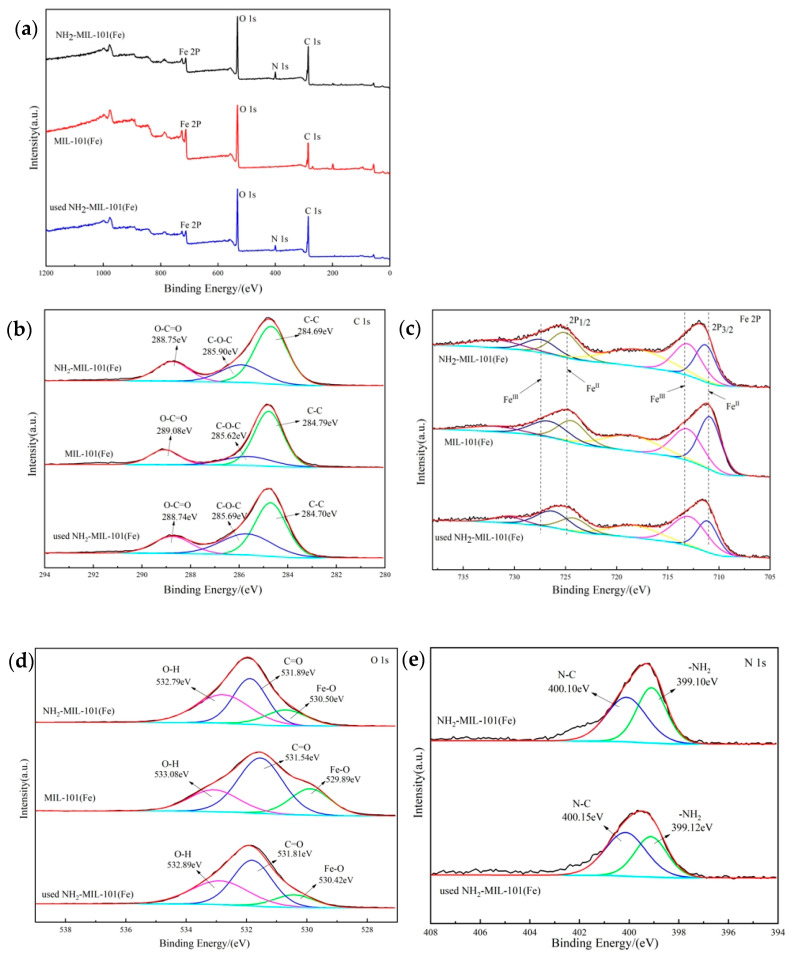
XPS spectra of NH_2_-MIL-101(Fe), MIL-101(Fe), and utilized NH_2_-MIL-101(Fe): (**a**) survey, (**b**) C 1s, (**c**) Fe 2p, (**d**) O 1s, and (**e**) N 1s.

The XPS images of the two catalysts are shown in Figure 4a; the occurrence of N1s spectra indicated the introduction of amino functional groups. The three characteristic peaks at 284.69 eV (C-C), 285.90 eV (C-O-C), and 288.75 eV (O-C=O) of the C 1s spectrum of Figure 4b were associated with the terephthalic acid group and carboxyl group [34].

The Fe 2p spectrogram showed two fitted peaks in Figure 4c; the corresponding peaks of Fe^3+^ were at 2p_1/2_ and 2p_3/2_ with binding energies of 727.44, 713.03 eV [35]; and the corresponding peaks of Fe^2+^ were at 2p_1/2_ and 2p_3/2_ with binding energies of 725.06, 711.31 eV [36,37], respectively. For the XPS spectrum of O 1s in Figure 4d, three peaks appearing at 530.50 eV, 531.89 eV, 532.79 eV corresponded to Fe-O, C=O, and O-H, respectively [38]. In Figure 4e, the N1s spectrum showed two binding energy peaks at 400.10 eV, 399.10 eV, corresponding to N-C and -NH_2_, respectively [39].

### 2.2. Degradation of OG under Different Conditions

#### 2.2.1. Effect of Different Systems, Initial pH, Temperature, Catalyst Dosage, and PMS Concentration on Degradation Experiments

**Figure 5:**
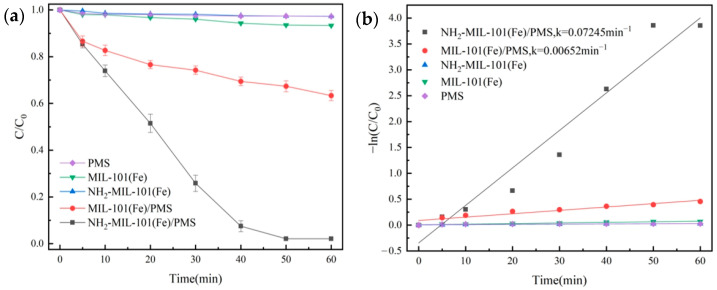
Removal efficiency of OG in different systems (**a**) and pseudo-first-order kinetic fitting curves (**b**).

The degradation experiments of OG under different systems are shown in Figure 5a, and quasi-primary kinetic curves were fitted, and the results are shown in Figure 5b. The NH_2_-MIL-101(Fe) and MIL-101(Fe) had weak adsorption effects (2.7% and 6.6%, respectively) for OG. The degradation rate of OG in the OG/PMS system was 2.8%, and it suggested that it was difficult only for self-decomposition of PMS to efficiently degrade OG.

The degradation rate of OG for the MIL-101(Fe)/PMS combination only could reach 36.6%; it indicated that MIL-101(Fe) had a restricted ability to activate PMS. For the NH_2_-MIL-101(Fe)/PMS system, due to the addition of the amino group, the degradation rate had a considerable improvement and could reach 97.9%, and its reaction constant *k* value was 0.07245 min^−1^. The above results showed that NH_2_-MIL-101(Fe) had a better catalytic performance. Introduction of the amino group enhanced the catalyst’s ability to activate the PMS [40], which led to the generation of radicals (SO_4_^•−^ and •OH) and non-radicals (^1^O_2_) in the system to obtain a strong degradation performance for organic pollutants.

**Figure 6:**
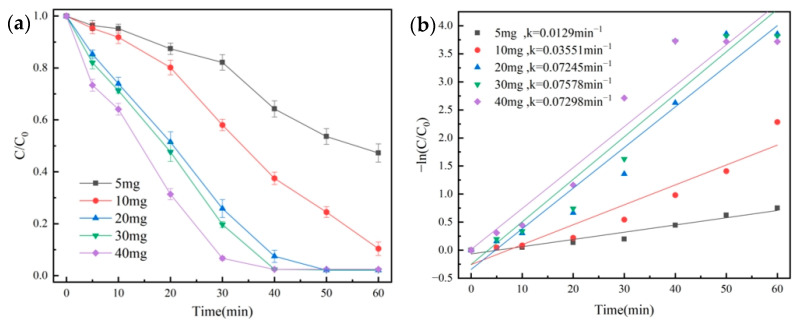
Removal efficiency of OG for catalyst dosage (**a**) and pseudo-first-order kinetic fitting curves (**b**).

The effect of different catalyst dosages on the degradation of OG using the NH_2_-MIL-101(Fe)/PMS system was investigated. We made up 100 mL of OG solution (50 mg/L), and then 1 mL of PMS solution (100 mmol/L) was added. Under the conditions of temperature at 25 °C and initial pH = 7.3, the degradation effect of OG was shown in Figure 6a, quasi-primary kinetic curves were fitted, and the results were shown in Figure 6b.

The *k* value increased from 0.0129 min^−1^ to 0.07298 min^−1^, and the degradation rate of OG increased from 52.7% to 97.6% when the dosage of the NH_2_-MIL-101(Fe) catalyst was raised from 5 mg to 40 mg. According to this, raising the dosage of the catalyst might offer more active sites and greatly speed up the rate that PMS was activated to produce more radicals. When the catalyst dosage was higher than 20 mg, a degradation rate of more than 97.6% was reached after 50 min. Therefore, the catalyst dosage in the subsequent trial was set to 20 mg to take the economy and degrading effect into account.

**Figure 7:**
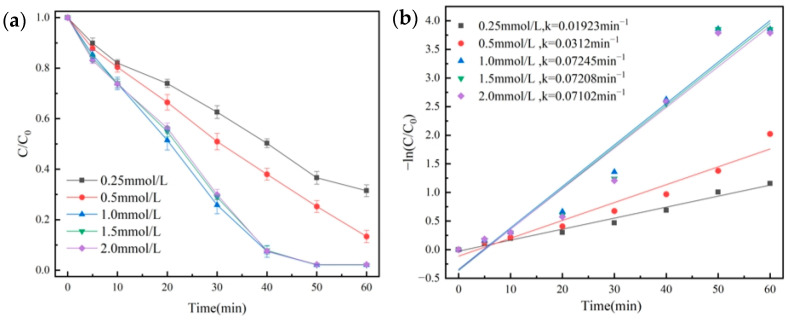
Removal efficiency of OG in PMS dosage (**a**) and pseudo-first-order kinetic fitting curves (**b**).

The effects of different PMS concentrations were investigated. We made up 100 mL of OG solution (50 mg/L), and then 20 mg of the catalyst was added. Under the conditions of temperature at 25 °C and initial pH = 7.3, the degradation effect of OG is shown in Figure 7a. Quasi-primary kinetic curves were fitted, and the results are shown in Figure 7b.

As shown in Figure 7a, when the PMS concentration increased from 0.25 mmol/L to 1.0 mmol/L, the OG degradation rate rose from 68.5% to 97.7%, and it showed a significant increase for the *k* value from 0.01923 min^−1^ to 0.07102 min^−1^. The results suggested that properly increasing the concentration of PMS was advantageous for increasing the contact between PMS and the catalyst. However, the excessive addition of PMS produced a lot of free radicals that competed with the target organics for SO_4_^•−^, so they caused a quenching reaction, which was why there was no increase in the dosage of PMS [41].

**Figure 8:**
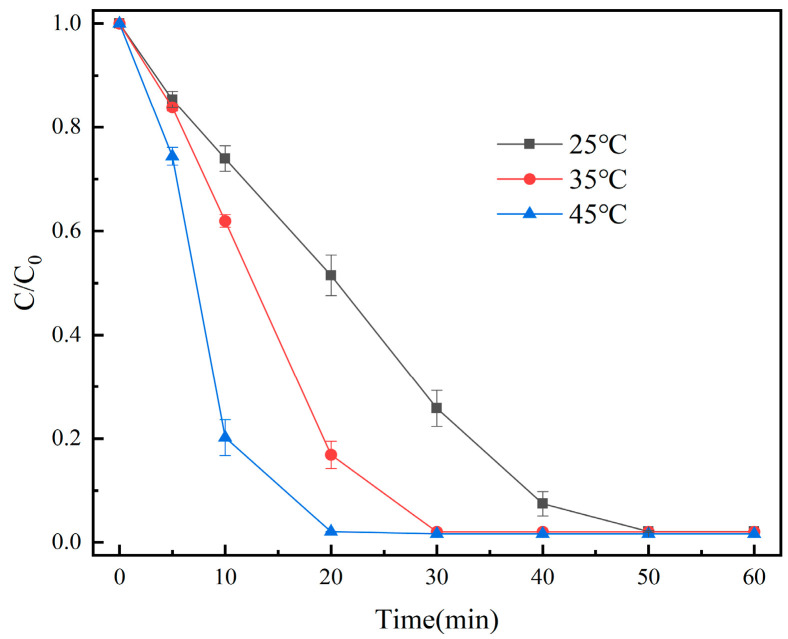
Removal efficiency of OG in different temperatures.

The effect of different temperatures (25 °C, 35 °C, and 45 °C) was investigated. We made up 100 mL of OG solution (50 mg/L), and then 20 mg of the catalyst and 1 mL of PMS solution (100 mmol/L) were added. Under the conditions of the initial pH = 7.3, the degradation effect of OG is shown in Figure 8.

As seen in Figure 8, the degradation rate of OG increased from 97.9% to 98.4% when the temperature was raised from 25 °C to 45 °C. The findings suggested that raising the temperature aided in the degradation of OG. More oxidative radicals were produced as a result of the temperature rise [42].

**Figure 9:**
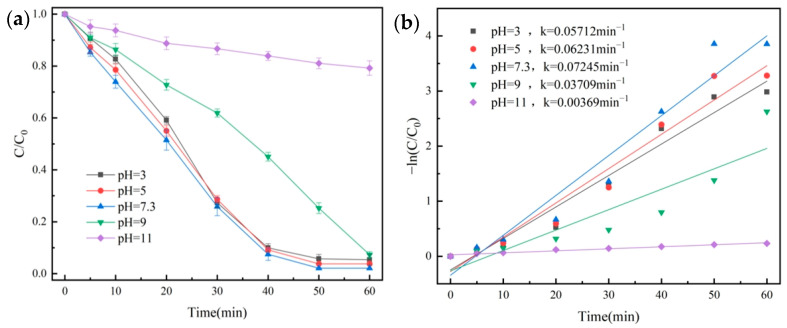
Removal efficiency of OG in various pH conditions (**a**) and pseudo-first-order kinetic fitting curves (**b**).

The effects of different initial pHs were investigated. We made up 100 mL of OG solution (50 mg/L), and then 20 mg of catalyst and 1 mL of PMS solution (100 mmol/L) were added. Under the conditions of temperature at 25 °C, the degradation effect of OG was shown in Figure 9a, quasi-primary kinetic curves were fitted, and the results are shown in Figure 9b.

As seen from Figure 9a, when the pH value was in the range of 3–9, the degradation rate of OG could reach up to 92.7% after 60 min. The highest degradation rate was 97.9% when the pH was 7.3. When the pH was 11, the degradation rate of OG was only 20.8% at 60 min, with a lower *k* value of 0.00369 min^−1^.

#### 2.2.2. Effect of Inorganic Anions on OG Degradation

**Figure 10:**
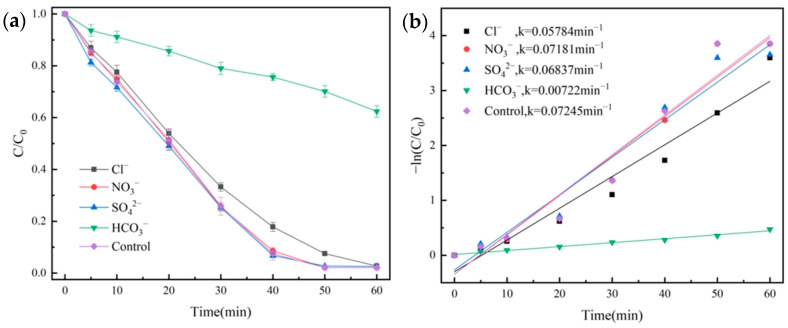
Influence of various anions on the degradation of OG (**a**) and pseudo-first-order kinetic fitting curves (**b**).

We added NaHCO_3_ (0.1680 g), Na_2_SO_4_ (0.2841 g), NaNO_3_ (0.1700 g), and NaCl (0.1169 g) to the NH_2_-MIL-101(Fe)/PMS system, the effect of different anions on the degradation of OG was investigated. We made up 100 mL of OG solution (50 mg/L), and then 20 mg of catalyst and 1 mL of PMS solution (100 mmol/L) were added. Under the conditions of temperature at 25 °C and pH = 7.3, the degradation effect of OG is shown in Figure 10a, quasi-primary kinetic curves were fitted, and the results are shown in Figure 10b.

As can be seen in Figure 10a, SO_4_^2−^ and NO_3_^−^ had little effect on the degradation, and Cl^−^ had a slight inhibitory effect on the degradation of OG. This was because Cl^−^ reacted with SO_4_^•−^ to form Cl^−•^ with lower oxidation potential [43]. In contrast, HCO_3_^−^ showed significant inhibition of the degradation effect, with a degradation rate of only 37.6% at 60 min, with a *k* value of 0.00722 min^−1^; its reason was that the addition of HCO_3_^−^ increased the pH of the solution, and HCO_3_^−^ also trapped reactive radicals, which thus inhibited the degradation effect [44].

#### 2.2.3. Catalyst Recycling Test

**Figure 11:**
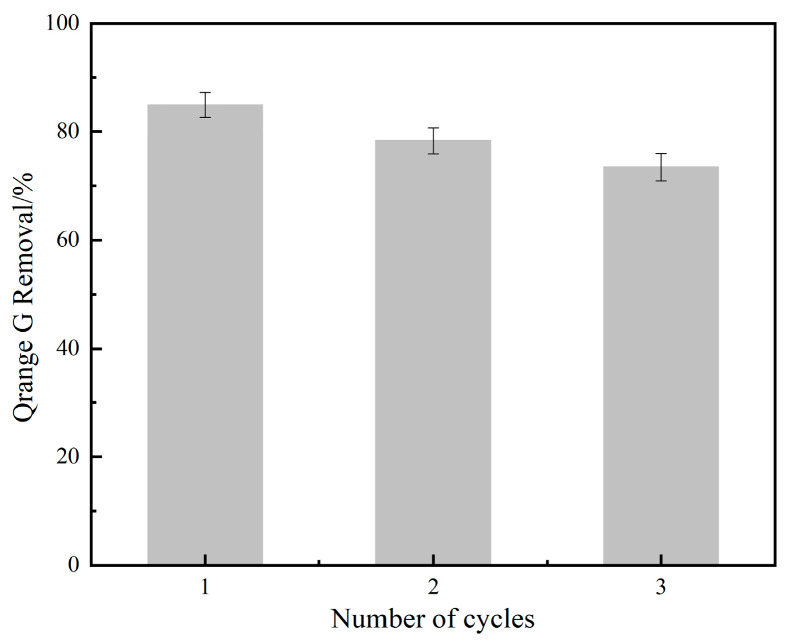
Recycling tests of NH_2_-MIL-101(Fe).

The recycling of NH_2_-MIL-101(Fe) was investigated. We made up 100 mL of OG solution (50 mg/L), and then 20 mg of catalyst and 1 mL of PMS solution (100 mmol/L) were added. Under the conditions of temperature at 25 °C and initial pH = 7.3, the results are shown in Figure 11. For three consecutive recycling experiments, the degradation rates of OG were 84.9%, 78.3%, and 73.1%, respectively, indicating that NH_2_-MIL-101(Fe) had some reusability.

#### 2.2.4. Active Radicals in Degradation

**Figure 12:**
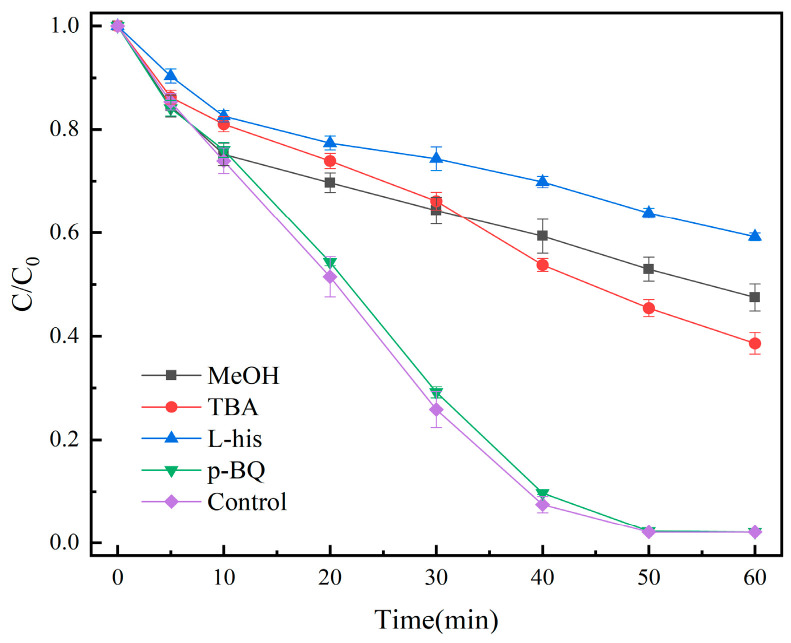
Effect of different quenchers on the removal of OG.

The free-radical species in the system were detected using quenching experiments. We made up 100 mL of OG solution (50 mg/L), and then 20 mg of catalyst and 1 mL of PMS solution (100 mmol/L) were added. Under the conditions of temperature at 25 °C and initial pH = 7.3, the results are shown in Figure 12.

Methanol (MeOH), tert-butanol (TBA), p-benzoquinone (p-BQ), and L-histidine (L-his) were used for the quenching experiments of SO_4_^•−^, •OH, •O_2_^−^, and ^1^O_2_ active species, respectively. The removal rate decreased to 52.5% and 61.4% when 0.1 mol MeOH and TBA were added, respectively, whereas the addition of 0.5 mmol p-BQ did not have any effect on the degradation; the addition of 0.5 mmol L-His decreased the removal rate to 40.8%, indicating the possible generation of SO_4_^•−^, •OH, and ^1^O_2_ involved in the degradation of OG.

#### 2.2.5. UV–Vis Spectrum of OG Solution in Different Stage

**Figure 13:**
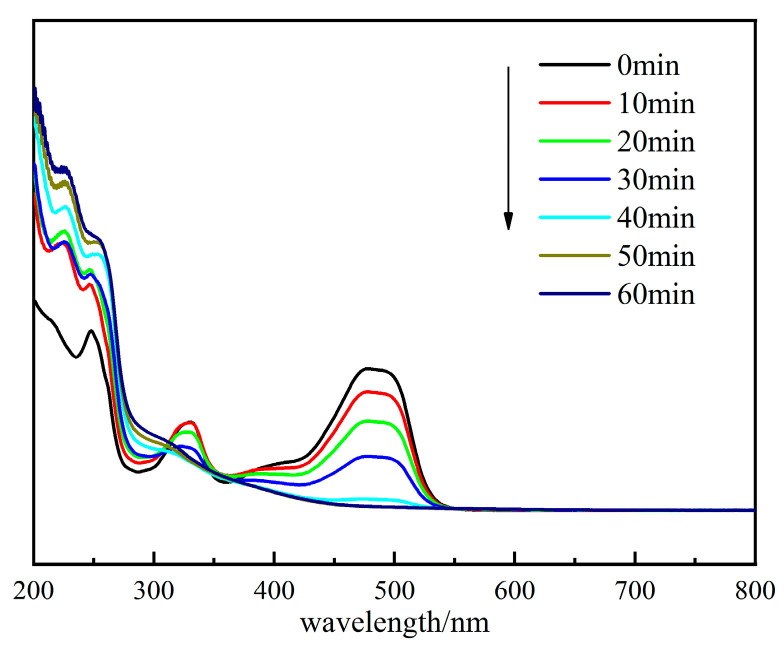
UV–Vis absorption spectra of OG solution during degradation.

UV full wavelength scanning of the sample solution was carried out at different reaction time points to observe the spectral changes of the OG solution and its by-products of degradation. We made up 100 mL of OG solution (50 mg/L), and then 20 mg of catalyst and 1 mL of PMS solution (100 mmol/L) were added. Under the conditions of temperature at 25 °C and initial pH = 7.3, the results are shown in Figure 13.

Figure 13 shows that the OG had a more pronounced absorption peak at 484 nm. As time passed, this characteristic peak first rapidly declined. The 333 nm peak was assigned to the naphthalene ring in the structure of OG [45]. It indicated that the azo bond and aromatic ring in the OG structure were disrupted by SO_4_^•−^,•OH, and ^1^O_2_, which were formed with the activation of PMS. 

#### 2.2.6. Performance Comparison of Different Advanced Oxidation Systems

The removal performance of OG for the NH_2_-MIL-101(Fe)/PMS system was compared with those of other advanced oxidation systems reported in the literature [18,19,46,47,48], and the results are shown in Table 1.

In comprehensive comparison, the NH_2_-MIL-101(Fe)/PMS system showed a high OG removal rate in a short period of time at a low catalyst dosage and PMS concentration. The above results also indicated that the system had good OG removal performance.

### 2.3. Reaction Mechanism Analysis

Based on the results of the above quenching experiments, it was inferred that the NH_2_-MIL-101(Fe)/PMS system produced SO_4_^•−^, •OH, and ^1^O_2_ to degrade OG, which mainly consisted of the following steps: the PMS molecule transferred the electrons using Fe^2+^ and activated it to produce SO_4_^•−^, while Fe^2+^ was oxidized to produce Fe^3+^ [49] (Equation (1)). In the presence of PMS, Fe^3+^ could be reduced to Fe^2+^ (Equation (2)). The SO_4_^•−^ produced could further react with H_2_O and OH^−^ to form •OH [50] (Equations (3) and (4)). The SO_5_^•−^ produced during the reaction would react with H_2_O to form ^1^O_2_ (Equation (5)), self-decomposition of PMS also produced SO_5_^2−^ which reacted with PMS to form ^1^O_2_ (Equations (6) and (7)). Amino functional groups with high electron density could provide electrons for Fe^3+^ to produce more Fe^2+^ and accelerate the Fe^3+^/Fe^2+^ cycle, and thus accelerated the decomposition of PMS [51] (Equations (8) and (9)).

(1) $${\mathrm{Fe}}^{2+}+{\mathrm{HSO}}_{5}^{-}\to {\mathrm{Fe}}^{3+}+{\mathrm{SO}}_{4}^{•-}+{\mathrm{OH}}^{-}$$

(2) $${\mathrm{Fe}}^{3+}+{\mathrm{HSO}}_{5}^{-}\to {\mathrm{Fe}}^{2+}+{\mathrm{SO}}_{5}^{•-}+H^{+}$$

(3) $${\mathrm{SO}}_{4}^{•-}+H_{2}O\to {\mathrm{SO}}_{4}^{2-}+•\mathrm{OH}+H^{+}$$

(4) $${\mathrm{SO}}_{4}^{•-}+{\mathrm{OH}}^{-}\to {\mathrm{SO}}_{4}^{2-}+•\mathrm{OH}$$

(5) 2SO5•−+H2O→2HSO4−+1.5O21

(6) $${\mathrm{HSO}}_{5}^{-}\to {\mathrm{SO}}_{5}^{2-}+H^{+}$$

(7) HSO5−+SO52−→HSO4−+SO42−+O21

(8) $${\mathrm{HSO}}_{5}^{-}\overset{-{\mathrm{NH}}_{2}}{\to }e^{-1}+H^{+}+{\mathrm{SO}}_{5}^{•-}$$

(9) $${\mathrm{Fe}}^{3+}+e^{-1}\to {\mathrm{Fe}}^{2+}$$

### 2.4. Pathway Analysis of OG Degradation

**Figure 14:**
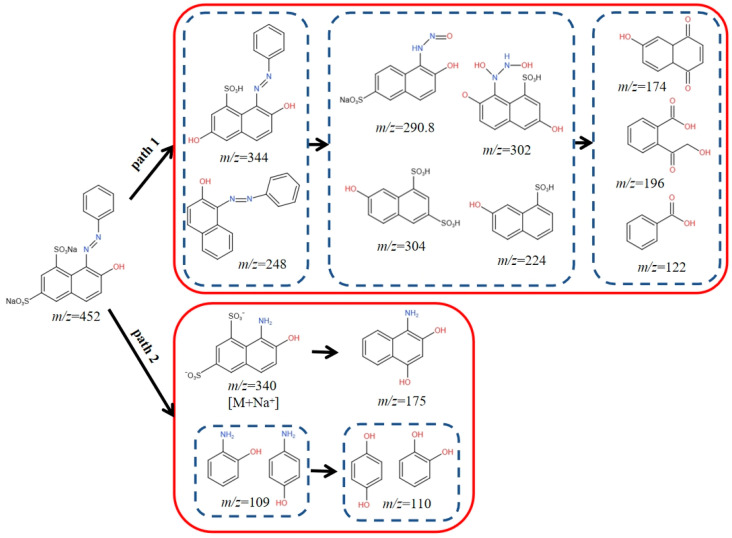
Pathway analysis of OG degradation.

To illustrate the possible OG degradation pathways, the degradation intermediates of OG were identified using LC-MS. It was found that there were fifteen intermediates. A plausible OG breakdown mechanism was conjectured (Figure 14).

In path 1, through an electrophilic substitution desulfurization reaction, SO_4_^•−^, •OH, and ^1^O_2_ oxidized the OG (*m*/*z* = 452) molecule and yielded two distinct types of by-products (*m*/*z* = 344 and 248) [18]. When the C–N bond on the benzene ring or naphthol ring was broken, two naphthol derivatives (*m*/*z* = 302, 290.8) or (*m*/*z* = 304, 224) were produced. Two naphthol derivatives continued to react to produce three intermediate products (*m*/*z* = 174, 196 and 122) [52].

In path 2, the azo bonds of OG were broken to produce intermediates (*m*/*z* = 109 and 340), which were further subject to desulfonation and hydroxylated to produce a naphthol derivative (*m*/*z* = 175) and hydroquinone or catechol (*m*/*z* = 110) [5]. The possible end products included CO_2_ and H_2_O.

## 3. Materials and Methods

### 3.1. Experimental Reagents

Iron chloride hexahydrate (FeCl_3_·6H_2_O) was purchased from Tianjin Damao Chemical Reagent Factory (Tianjin, China). Orange G (OG), Terephthalic acid (H_2_BDC), 2-Aminoterephthalic acid (NH_2_-H_2_BDC), N,N-dimethylformamide (DMF), Potassium peroxymonosulfate (PMS), Sodium nitrate (NaNO_3_), Sodium bicarbonate (NaHCO_3_), Tert-butyl alcohol (TBA), Methanol (MeOH), P-Benzoquinone (P-BQ), and L-histidine (L-His) were obtained from Macklin (Shanghai, China). Sodium sulfate (Na_2_SO_4_), Sodium hydroxide (NaOH), and Concentrated sulfuric acid (H_2_SO_4_) were purchased from Xilong Science and Technology Co. (Shantou, China). Sodium chloride (NaCl) was supplied by Sinopharm Chemical Reagent (Shanghai, China). All the above chemical reagents were analytically pure except methanol which was chromatographic grade.

### 3.2. Preparation of MIL-101(Fe) and NH_2_-MIL-101(Fe)

In total, 2.5 mmol (0.6758 g) iron chloride hexahydrate (FeCl_3_·6H_2_O) and 1.24 mmol (0.2060 g) terephthalic acid (H_2_BDC) were added to 15 mL N,N-dimethylformamide (DMF) solution. It was continuously stirred in a 60 °C water bath for 30 min until the solution was evenly mixed; the mixed solution was transferred to a Polytetra-fluoroethylene reactor; and the mixture was reacted for 20 h at 110 °C. Finally, the crystalline product was washed with DMF, ethanol, and deionized water, respectively, for 3–5 times to remove any unreacted organic ligands. We dried the product in a drying oven at 60 °C for 18 h; it was ground and sieved through a 100-mesh sieve to obtain a brown catalyst. NH_2_-MIL-101(Fe) was prepared in essentially the same way as described above by replacing 1.24 mmol (0.2060 g) of H_2_BDC with 1.24 mmol (0.2246 g) of NH_2_-H_2_BDC.

### 3.3. Characterization Methods

The structural information of NH_2_-MIL-101(Fe) was obtained using Fourier Transform Infrared (FTIR) (Nicolet 330, Waltham, MA, USA) and X-ray diffraction (XRD) (Rigaku Smartlab 9 KW, Tokyo, Japan). The morphology was observed using scanning electron microscopy (SEM) (ZEISS Sigma 300, Oberkochen, Germany). The chemical composition and elemental valence states was analyzed with an X-ray Photoelectron Spectrometer (XPS) (Thermo Scientific K-Alpha, Waltham, MA, USA). The absorbance of OG solution was determined using a UV–Vis spectrophotometer (UV-2600i, Tokyo, Japan). The degradation intermediates of OG were determined using LC-MS. Detailed information of LC-MS is given in the Supplementary Material (S1).

### 3.4. Degradation Tests

The degradation experiments were carried out in a water-bath thermostatic oscillator at a temperature of 25 °C and a rotation rate of 150 rpm. The pH of the initial solution was adjusted using 0.1 mol/L of sodium hydroxide or sulfuric acid. We measured 100 mL of OG solution with a concentration of 50 mg/L in a glass conical flask, followed by the addition of solid catalytic material and PMS solution, and then we started timing. The filtrate was obtained after filtering the sample solution through a 0.45 μm membrane at a fixed time point, and the absorbance of OG solution was determined using a UV–Vis spectrophotometer at a wavelength of 484 nm.

### 3.5. Regeneration Performance Test of Catalyst

The material was separated with a 0.45 μm filter membrane after one degradation experiment, and the solids on the membrane were rinsed with anhydrous ethanol and deionized water multiple times. The catalyst was then placed in a drying oven at 60 °C for 12 h in order to examine the regeneration performance of the catalyst.

## 4. Conclusions

The catalyst NH_2_-MIL-101(Fe) was prepared using the solvothermal approach. We selected the OG as the target pollutant for degradation, and we studied the degradation performance and the influencing factors of degradation process. The main results were as follows:

1. With 100 mL of OG solution (50 mg/L), 20 mg of catalyst, and 1 mL of PMS solution (100 mmol/L) were added. Under the conditions of 25 °C and pH = 7.3, the degradation rate of OG with the MIL-101(Fe) system was only 36.6% within 60 min, while the NH_2_-MIL-101(Fe)/PMS system could reached 97.9%. The improved system had a good effect in the pH range of 3~9, with a degradation rate of more than 92.7%.
2. In the anion interference experiments, SO_4_^2−^ and NO_3_^−^ did not significantly inhibit the degradation experiments, except for Cl^−^ and HCO_3_^−^. The free-radical quenching experiments showed that the NH_2_-MIL-101(Fe)/PMS system produced three kinds of reactive substances, SO_4_^•−^, •OH, and ^1^O_2_. The degradation rate of OG was still more than 73.1% with the recycling of NH_2_-MIL-101(Fe) three times.
3. In a shorter time, the NH_2_-MIL-101(Fe)/PMS system was able to efficiently degrade the OG in comparison to other advanced oxidation technologies. This work supported the investigation into the impact of adding amino functional groups in PMS activation for the breakdown of azo fuel. It is anticipated that the technology will also be used to treat the actual wastewater of azo dyes.

## Figures and Tables

**Table 1:** Degradation comparison of OG with different catalyst/oxidant systems.

Catalysts	Dosage	Oxidizing Agent and Concentration	Added Energy	V_OG_, C_OG_	Removal Rate and Time	The Literature
Fe_3_O_4_/CeO_2_-OX	2.0 g/L	H_2_O_2_, 26 mmol	-	50 mL, 50 mg/L	98.2%, 120 min	[46]
MnFe_2_O_4_/α-MnO_2_ (1:9)	0.1 g/L	PMS, 1000 mg/L	-	50 mL, 50 mg/L	96.8%, 30 min	[18]
Sepiolite-TiO_2_ NCs	0.8 g/L	-	UV 300 W	-, 10 mg/L	98.8%, 150 min	[47]
Sn/TiO_2_/AC	12.5 g/L	H_2_O_2_, 1.5 mL/L	UV 300 W	2500 mL, 50 mg/L	99.1%, 60 min	[48]
MIL-53(Fe)	1.0 g/L	PS, 32 mmol	-	-, 0.2 mmol	93.7%, 180 min	[19]
NH_2_-MIL-101(Fe)	0.2 g/L	PMS, 0.1 mmol	-	100 mL, 50 mg/L	97.9%, 60 min	This work

Note: “-” indicates no data.

## Data Availability

The data in this study are available upon reasonable request.

## Supplementary Materials

The following supporting information can be downloaded at: https://www.mdpi.com/article/10.3390/molecules29071488/s1, Figure S1: Secondary mass spectra of possible intermediates of OG (a–h); Table S1: Possible intermediates of OG.
