# Supplementary material for: Degradation of Orange G Using PMS Triggered by NH2-MIL-101(Fe): An Amino-Functionalized Metal–Organic Framework
Source: Molecules. 2024 Mar 27;29(7):1488. doi: 10.3390/molecules29071488 (PMC11013255; doi:10.3390/molecules29071488)
Supplement: Supplementary file 1 [file molecules-29-01488-s001.zip › molecules-2839235-supplementary.pdf]

Supporting Information

LC-MS detection information

Liquid phase conditions:
Instrumentation: waters HClass
mobile phase: 90 per cent acetonitrile (with 0.1 per cent formic acid)
column temperature: 40℃
flow rate：0.6mL/min

Mass Spectrometry Conditions：
Instrumentation: waters G2-XS Qtof
Positive mode： Voltage 3.5kv
Ion source temperature 110℃
Desolventisation temperature 400℃
Nitrogen flow rate 800L/h
Negative mode： Voltage 3kv
Ion source temperature 110℃
Desolventisation temperature 400℃
Nitrogen flow rate 800L/h

Table S1: Possible intermediates of OG

NO.	Chemical structure	Molecular formula	m/z
1	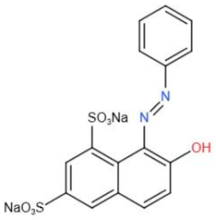	$C_{16}H_{10}N_2Na_2O_7S_2$	452
2	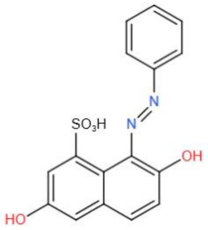	$C_{16}H_{12}N_2O_5S$	344
3	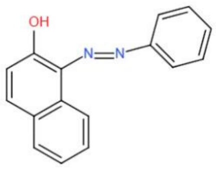	$C_{16}H_{12}ON_2$	248
4	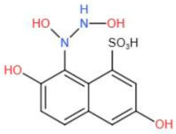	$C_{10}H_{10}O_7N_2S$	302
5	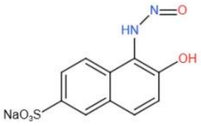	$C_{10}H_7N_2NaO_5S$	290.8
6	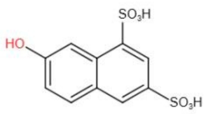	$C_{10}H_8O_7S_2$	304
7	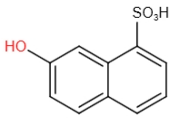	$C_{10}H_8O_4S$	224
8	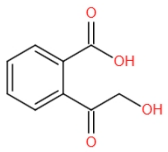	$C_9H_8O_5$	196

9	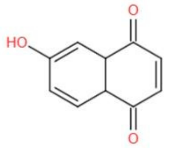	$C_{10}H_6O_3$	174
10	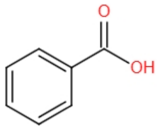	$C_7H_6O_2$	122
11	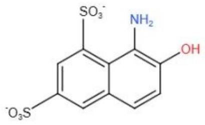	$C_{10}H_7O_7NS_2$	340
12	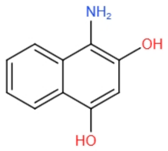	$C_{10}H_9O_2N$	175
13	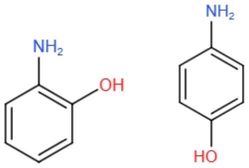	C_6H_6O	109
14	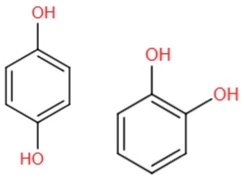	$C_6H_6O_2$	110

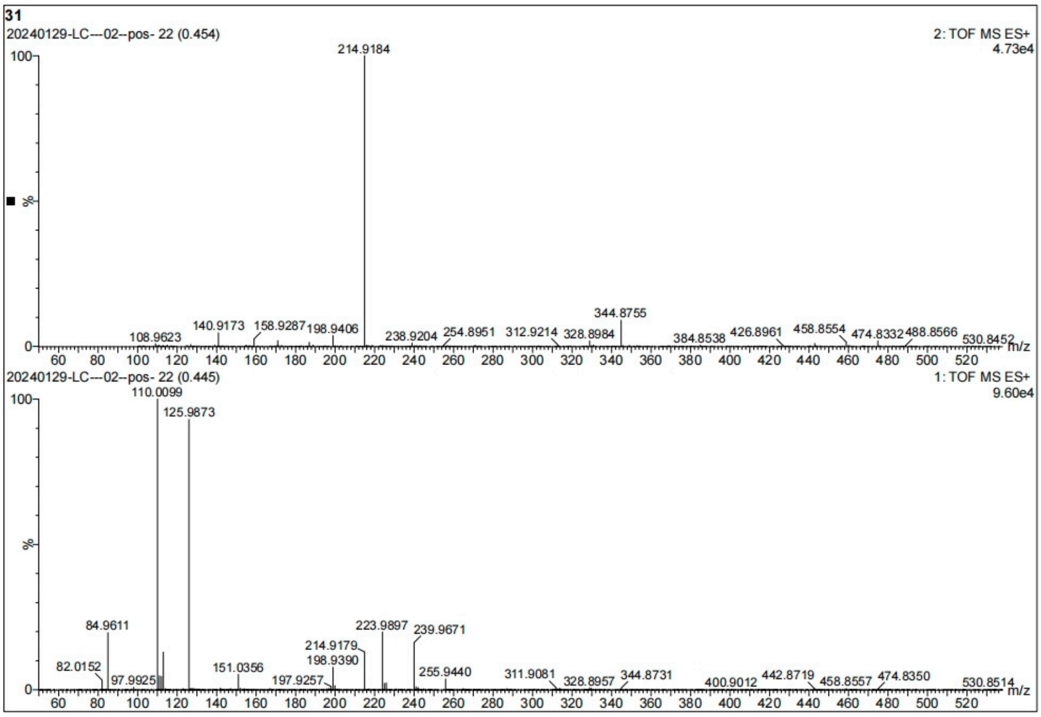
31
20240129-LC---02--pos- 22 (0.454)
2: TOF MS ES+
4.73e4
214.9184
108.9623
140.9173
158.9287
198.9406
238.9204
254.8951
312.9214
328.8984
344.8755
384.8538
426.8961
458.8554
474.8332
488.8566
530.8452
m/z
20240129-LC---02--pos- 22 (0.445)
1: TOF MS ES+
9.60e4
110.0099
125.9873
84.9611
82.0152
97.9925
151.0356
197.9257
198.9390
214.9179
223.9897
239.9671
255.9440
311.9081
328.8957
344.8731
400.9012
442.8719
458.8557
474.8350
530.8514
%

(a)

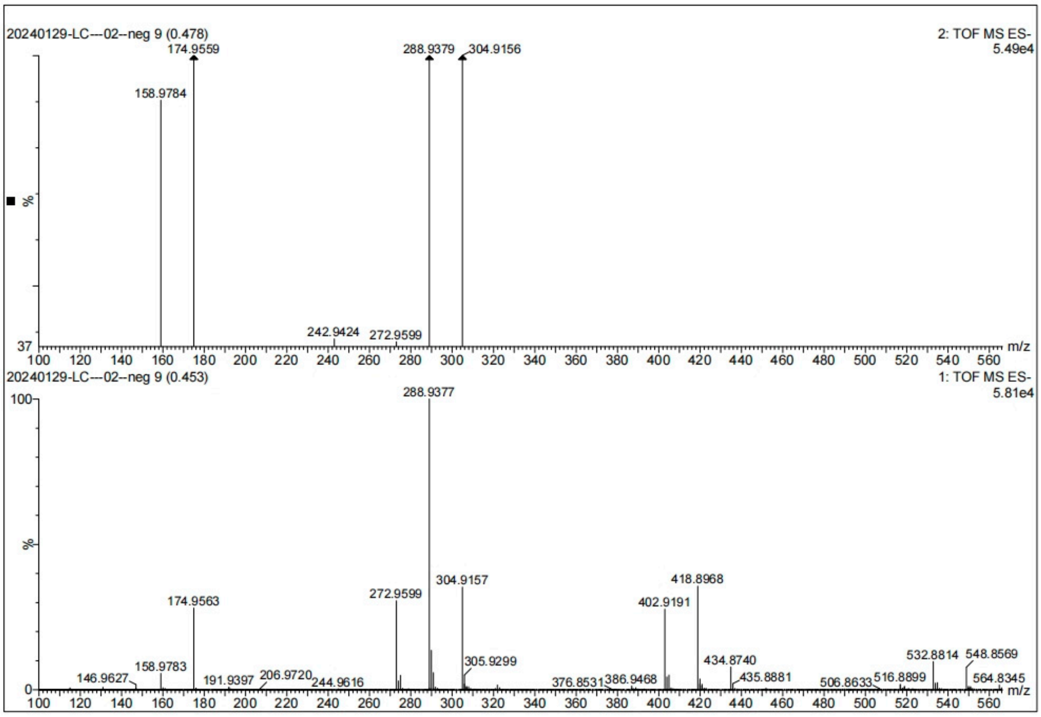
20240129-LC---02--neg 9 (0.478)
2: TOF MS ES-
5.49e4
174.9559
288.9379
304.9156
158.9784
242.9424
272.9599
m/z
20240129-LC---02--neg 9 (0.453)
1: TOF MS ES-
5.81e4
288.9377
304.9157
418.8968
402.9191
174.9563
272.9599
146.9627
158.9783
191.9397
206.9720
244.9616
305.9299
376.8531
386.9468
434.8740
435.8881
506.8633
516.8899
532.8814
548.8569
564.8345
%

(b)

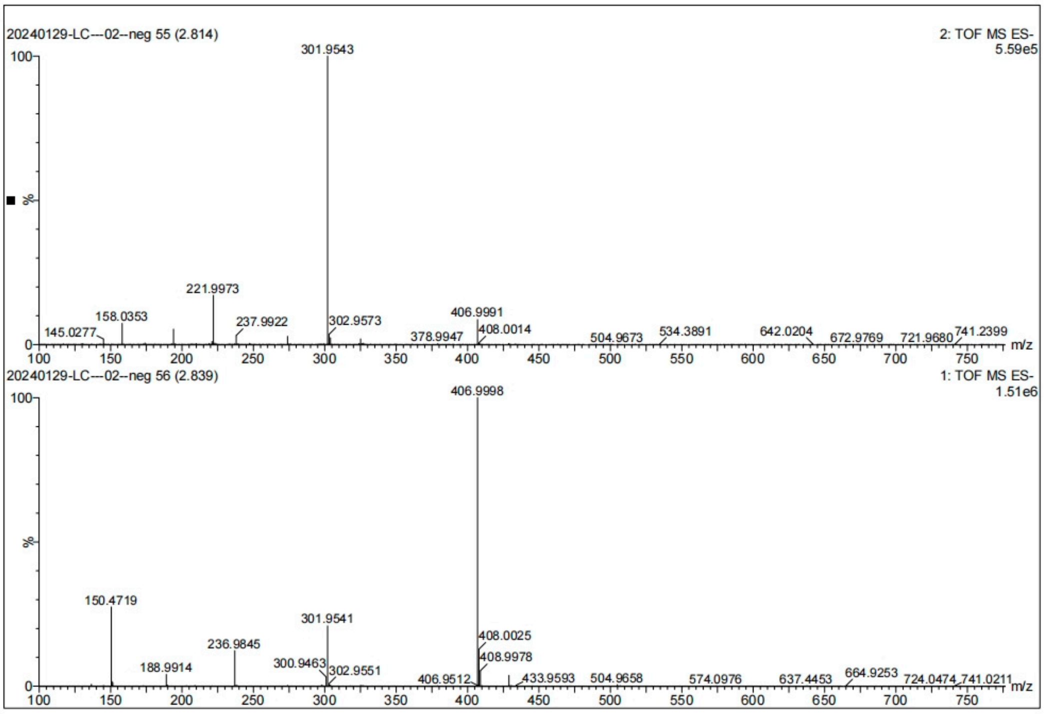
20240129-LC---02--neg 55 (2.814)
2: TOF MS ES-
5.59e5
301.9543
221.9973
158.0353
145.0277
237.9922
302.9573
378.9947
406.9991
408.0014
504.9673
534.3891
642.0204
672.9769
721.9680
741.2399
m/z
20240129-LC---02--neg 56 (2.839)
1: TOF MS ES-
1.51e6
406.9998
150.4719
301.9541
236.9845
188.9914
300.9463
302.9551
406.9512
408.0025
408.9978
433.9593
504.9658
574.0976
637.4453
664.9253
724.0474
741.0211
m/z
%
100
0

(c)

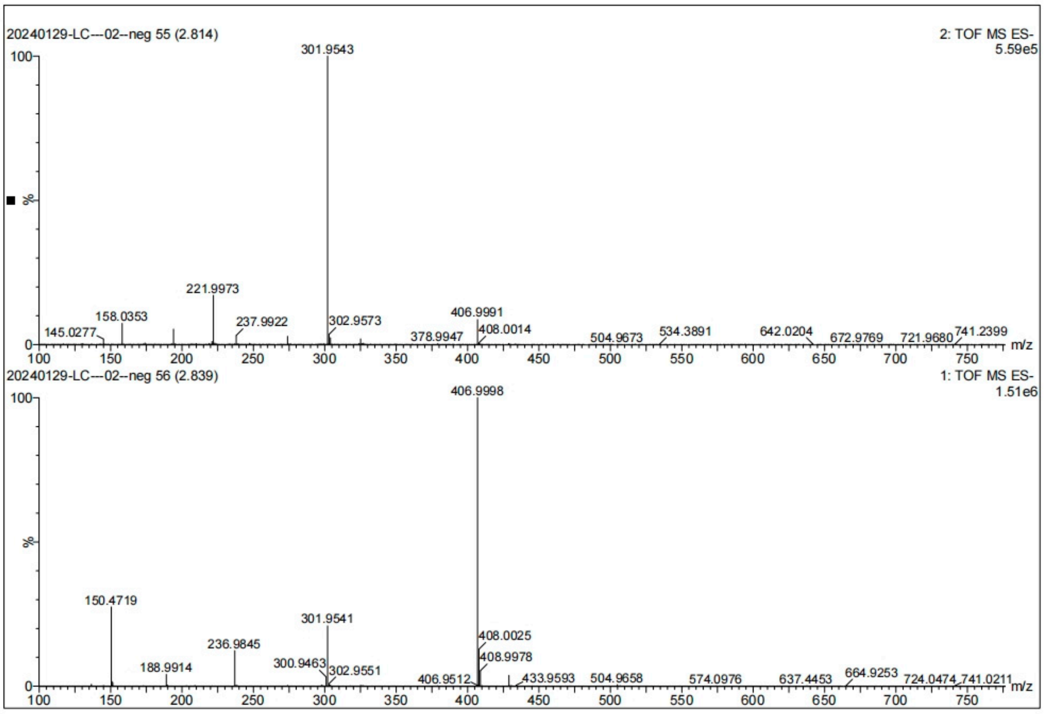
20240129-LC---02--neg 55 (2.814)
2: TOF MS ES-
5.59e5
301.9543
221.9973
158.0353
145.0277
237.9922
302.9573
378.9947
406.9991
408.0014
504.9673
534.3891
642.0204
672.9769
721.9680
741.2399
m/z
20240129-LC---02--neg 56 (2.839)
1: TOF MS ES-
1.51e6
406.9998
150.4719
301.9541
236.9845
188.9914
300.9463
302.9551
406.9512
408.0025
408.9978
433.9593
504.9658
574.0976
637.4453
664.9253
724.0474
741.0211
m/z
%
100
0

(d)

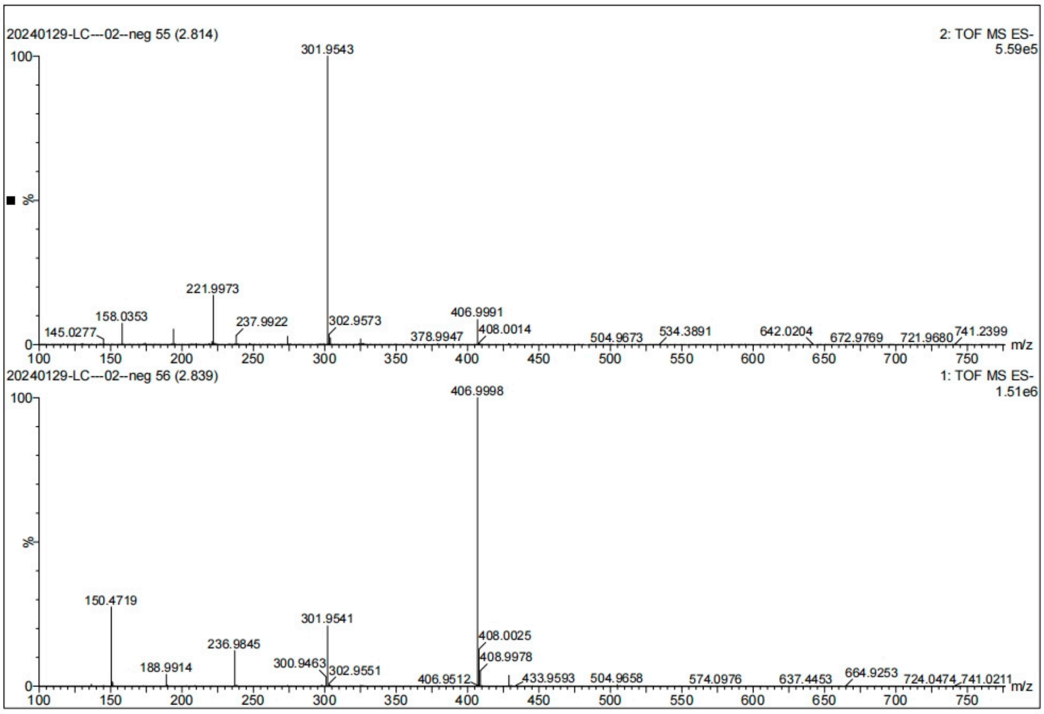
20240129-LC---02--neg 55 (2.814)
2: TOF MS ES-
5.59e5
301.9543
221.9973
158.0353
145.0277
237.9922
302.9573
406.9991
408.0014
378.9947
504.9673
534.3891
642.0204
672.9769
721.9680
741.2399
m/z
%
20240129-LC---02--neg 56 (2.839)
1: TOF MS ES-
1.51e6
406.9998
150.4719
301.9541
236.9845
188.9914
300.9463
302.9551
408.0025
408.9978
406.9512
433.9593
504.9658
574.0976
637.4453
664.9253
724.0474
741.0211
m/z
%

(e)

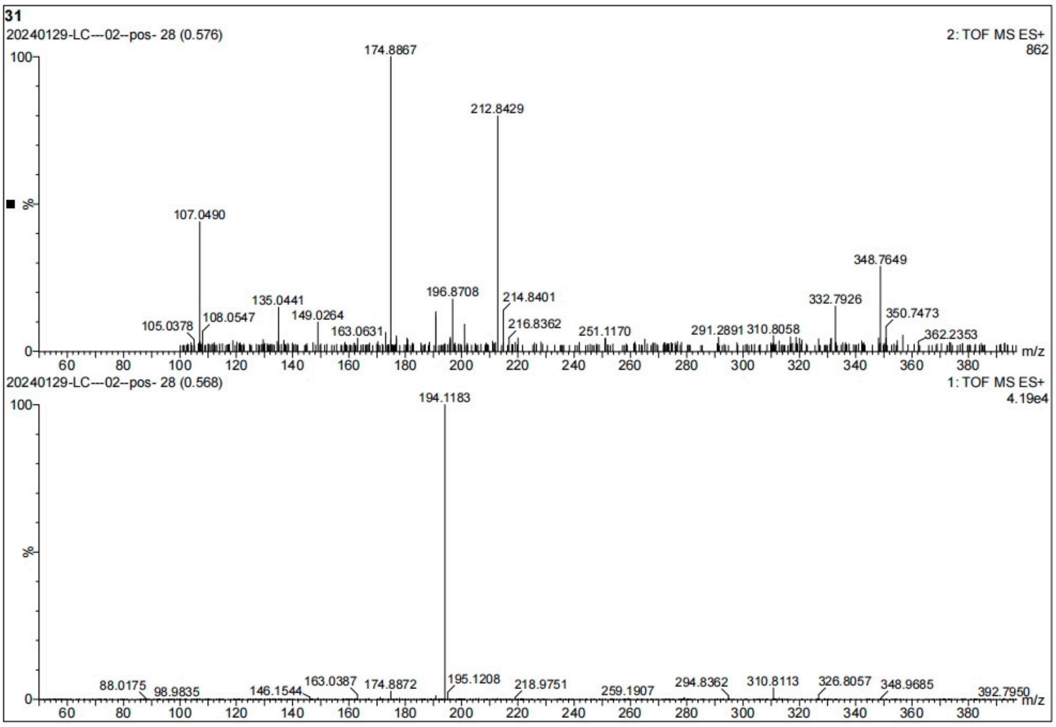
31
20240129-LC---02--pos- 28 (0.576)
2: TOF MS ES+
862
174.8867
212.8429
107.0490
348.7649
196.8708
214.8401
135.0441
332.7926
105.0378
108.0547
149.0264
163.0631
216.8362
251.1170
291.2891
310.8058
350.7473
362.2353
m/z
%
20240129-LC---02--pos- 28 (0.568)
1: TOF MS ES+
4.19e4
194.1183
88.0175
98.9835
146.1544
163.0387
174.8872
195.1208
218.9751
259.1907
294.8362
310.8113
326.8057
348.9685
392.7950
m/z
%

(f)

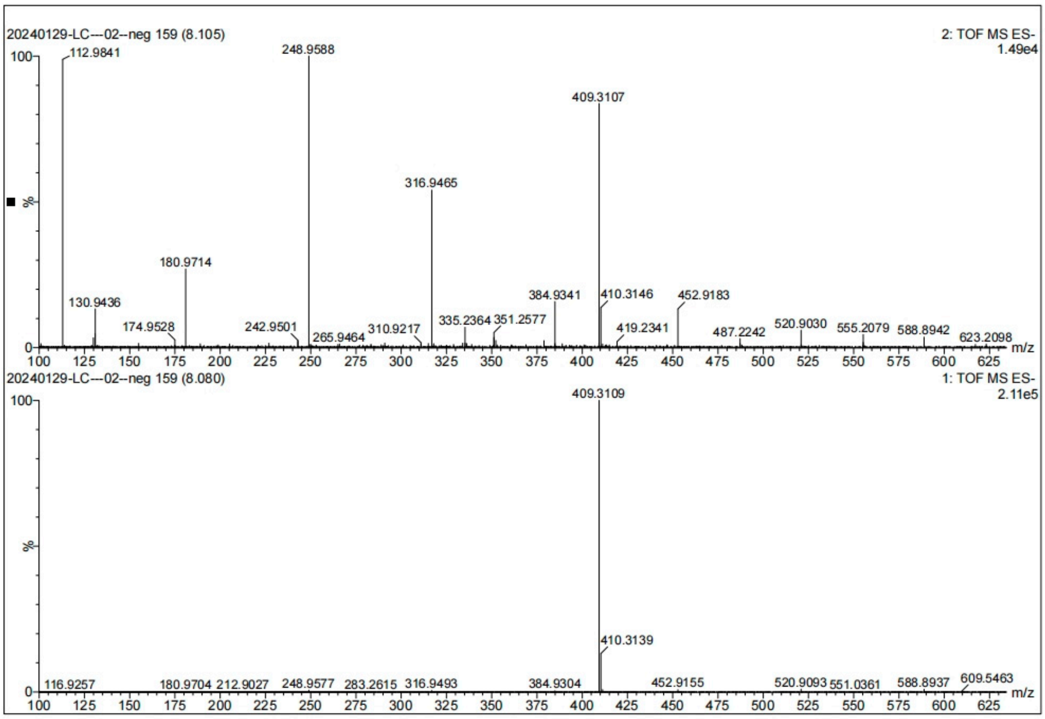

(g)

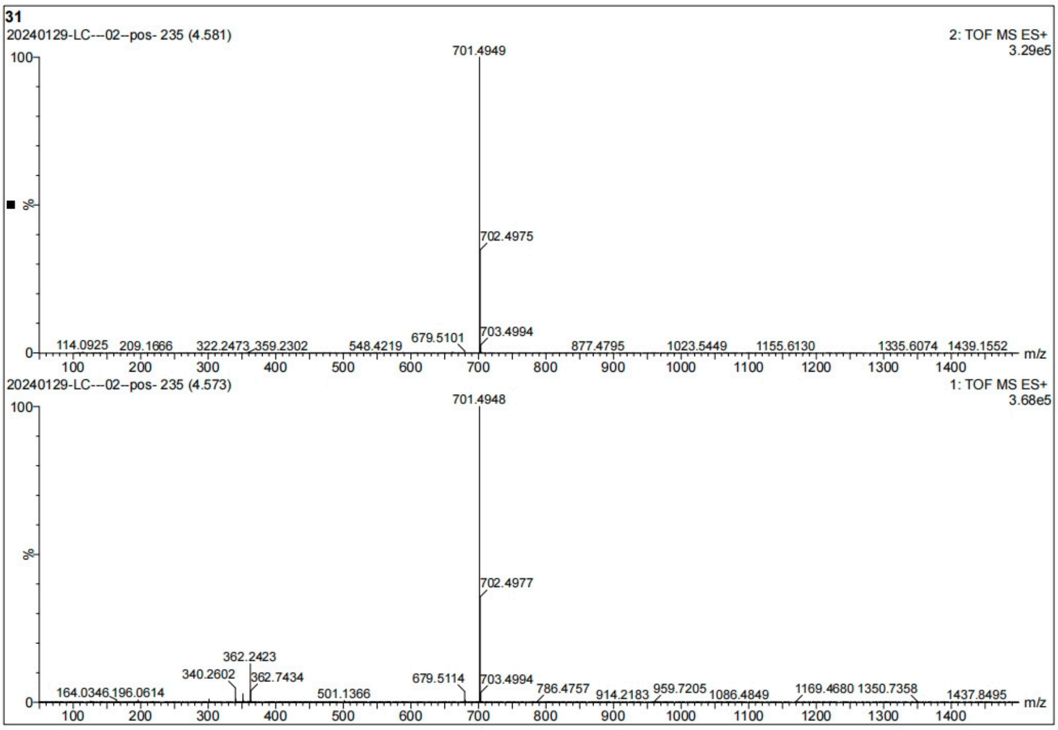

(h)

Figure S1: Secondary mass spectra of possible intermediates of OG(a-h)
